# Autoimmune Nodopathy With Anti-Neurofascin 186 Antibody

**DOI:** 10.7759/cureus.96112

**Published:** 2025-11-04

**Authors:** Ashik Anilkumar, Parameswaran Krishnan

**Affiliations:** 1 Neurology, Indo American Hospital Brain and spine Center, Vaikom, IND; 2 Neurology, Indo American Hospital Brain and Spine Center, Vaikom, IND

**Keywords:** anti-neurofascin 186 antibody, autoantibody profiling, autoimmune nodopathy, immune-mediated neuropathy, immunotherapy, rituximab therapy

## Abstract

Autoimmune neuropathy is a distinct subclass of immune-mediated neuropathy with a unique antibody-mediated profile. Early recognition is crucial for relief from the symptoms. The aim of this report was to highlight the clinical presentation and diagnostic and therapeutic challenges in a case of anti-neurofascin 186 nodopathy. A 46-year-old woman presented with progressive weakness in the lower limbs and pain in the palms and soles lasting for two years. She failed to respond to intravenous immunoglobulin and tapering steroids. Later, she was started on rituximab and showed significant neurological improvement over time. The findings indicate that early antibody testing can guide the effective management of autoimmune nodopathy.

## Introduction

In the broad spectrum of neuropathy, autoimmune neuropathy has a unique and increasingly recognized niche. Unlike metabolic and genetic neuropathies, autoimmune forms such as chronic inflammatory demyelinating polyneuropathy, Guillain-Barré syndrome (GBS), and the recently defined autoimmune neuropathy are associated with immune attacks on nerve components, particularly nodal and paranodal proteins such as neurofascin-155, neurofascin-186, contactin-1, and CASPR-1 [1]. While acute inflammatory demyelinating polyradiculoneuropathy (AIDP)/GBS typically responds well to intravenous immunoglobulin (IVIG) or plasmapheresis, patients with autoimmune nodopathies often show a poor response to IVIG and corticosteroids, requiring alternative immunotherapies such as rituximab [1].

We describe here a case of anti-neurofascin 186 nodopathy in a patient who initially presented with severe erythromelalgia and erythrodysesthesia of the soles and palms. The diagnostic challenges were mainly due to the similar presentation of the condition to that of other neuropathies. This case reinforces the need for early antibody screening and the initiation of individualized immunotherapy as early as possible.

## Case presentation

**Figure 1:**
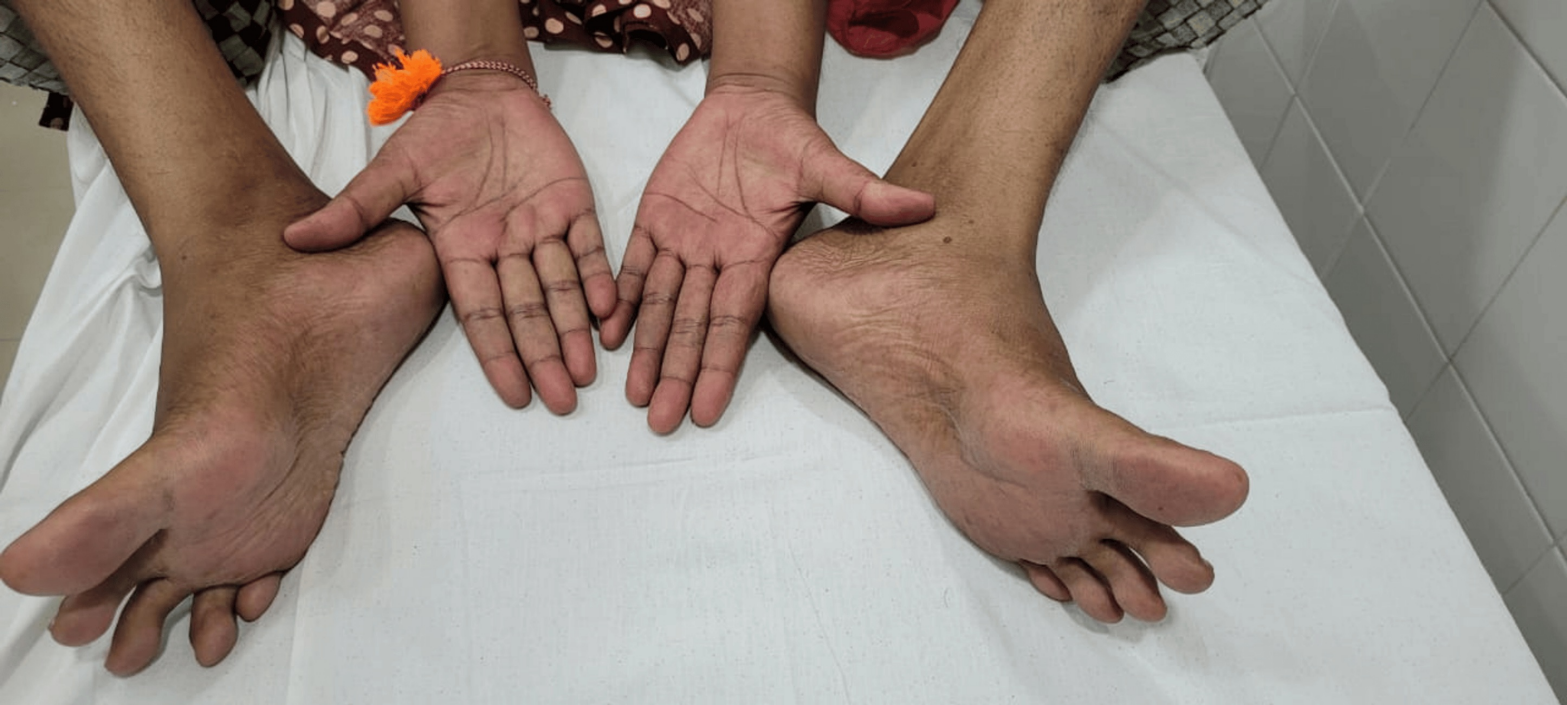
Clinical evidence of distal neuropathy in NF 186 autoantibody-mediated disease The distal muscle wasting of the hands and feet demonstrated chronic denervation changes in the limbs.

A 46-year-old woman presented to the neurology outpatient department with a two-year history of pain in the palms and soles. On examination, she was conscious, oriented, and hemodynamically stable (pulse: 82 beats/min; blood pressure: 120/90 mmHg; temperature: 98.6°F). A neurological examination revealed normal muscle strength (5/5) in the upper limb and reduced muscle strength (4/5) in the lower limbs. Sensations for crude touch, vibration, and proprioception were diminished, and deep tendon reflexes were reduced in the lower limb (1+). Distal wasting of the small muscles of the hands and feet was observed and, notably, dysesthesia in the palms and soles (Figure 1).

Laboratory evaluation demonstrated a normal hemogram, normal serum electrolytes, and normal liver, renal, and thyroid function tests. These findings effectively excluded metabolic, uremic, and hepatic neuropathies. Serum creatine phosphokinase levels were within the normal range, thereby ruling out inflammatory myopathies like dermatomyositis and polymyositis (Table 1).

**Table 1:** Routine blood investigations g/dl: grams per deciliter; µl: microliter; mg: milligram; mEq/l: milliequivalents per liter; dl: deciliter; IU: international units; U: units; AST: aspartate aminotransferase test; SGOT: serum glutamic-oxaloacetic transaminase test; ALT: alanine transaminase; SGPT: serum glutamic-pyruvic transaminase; ng: nanogram; CPK: creatinine phosphokinase; T: triiodothyronine; TSH: thyroid-stimulating hormone.

Investigation	Result	Reference range
Hemoglobin	12.4 g/dl	Male: 13.5-17.5 g/dl; female: 12.0-15.5 g/dl
Total leukocyte count	1,200/µl	4,000-11,000/µl
Differential count - neutrophils	86%	40%-70%
Differential count - lymphocytes	12%	20%-40%
Differential count - eosinophils	2%	1%-6%
Erythrocyte sedimentation rate	23 mm/first hour	Male: 0-15 mm/h; female: 0-20 mm/h
Random blood sugar	174 mg/dl	
Blood urea creatinine	9 mg/dl	7-20 mg/dl
Serum creatinine	0.8 mg/dl	Adult men: 0.7-1.3 mg/dl; adult women: 0.6-1.1 mg/dl; children: 0.3-0.7 mg/dl; infants: 0.2-0.4 mg/dl; newborns (up to 1 week): 0.3-1.0 mg/dl
Sodium	139 mEq/l	135-145 mEq/l
Potassium	3.8 mEq/l	3.5-5.0 mEq/l
Alkaline phosphatase	50 IU/l	
Serum bilirubin total	0.7 g/dl	0.3-1.2 g/dl
Total protein	6.7 g/dl	6.0-8.3 g/dl
Serum albumin	3.8 g/dl	3.5-5.0 g/dl
Serum globulin	2.9 g/dl	2.0-3.5 g/dl
AST (SGOT)	42 U/l	10-40 U/l
ALT (SGPT)	61 U/l	7-56 U/l
CPK	50 U/l	Male: 52-336 U/l; female: 38-176 U/l
Total T3	102 ng/dl	80-200 ng/dl
Total T4	71	5.0-12.0
TSH	0.35 IU/ml	0.4-4.0 IU/ml

The infectious workup, including hepatitis B surface antigen, human immunodeficiency virus (HIV), and venereal disease research laboratory test, was negative. Autoimmune screening, including anti-nuclear antibody, anti-thyroid peroxidase, and anti-thyroglobulin antibodies, was unremarkable, so systemic autoimmune disorders and autoimmune thyroiditis were unlikely (Table 2).

**Table 2:** Autoimmune screening tests for systemic autoimmune disorders anti-U1 RNP: anti-U1 ribonucleoprotein; anti-Ro/SSA: anti-Sjogren’s syndrome-related antigen A autoantibody; anti-La/SSB: anti-Sjogren’s syndrome-related antigen B; anti-Scl-70: anti-scleroderma 70; anti-Jo-1: anti-histidyl TRNA synthetase antibody; anti PM-Scl: anti-polymyositis-scleroderma antibody; anti-PCNA: autoantibody to proliferating-cell nuclear antigen; anti-ds DNA: antibody to double-stranded DNA.

Serial no	Test	Patient result
1	Anti-Smith	Negative
2	Anti-U1 RNP	Negative
3	Anti-Ro/SSA	Negative
4	Anti-La/SSB	Negative
5	Anti-Scl-70	Negative
6	Anti-centromere	Negative
7	Anti-Jo-1	Negative
8	Anti PM-Scl	Negative
9	Anti-nucleosome	Negative
8	Anti-histone	Negative
9	Anti-ribosomal P protein	Negative
10	Anti-mitochondrial	Negative
11	Anti-PCNA	Negative
12	Anti-thyroglobulin antibody	<1.30 U/ml (<20-40: normal; ≥40: positive)
13	Anti-thyroid peroxidase antibody	<28 U/ml (<35: normal; ≥35: positive)
14	Anti-ds DNA antibody	6.40 U/ml (<16: normal; ≥16: positive)

**Figure 2:**
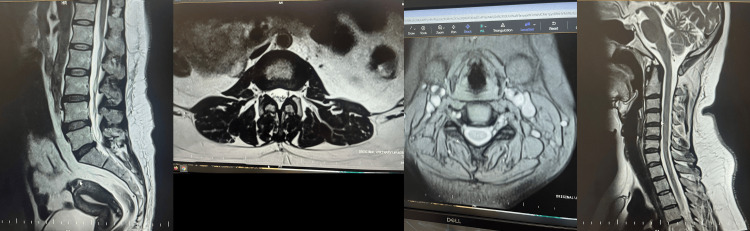
T1-weighted axial and sagittal sequences No dorsal nerve root thickening was noted in the cervical and dorsal lumbar region.

The nerve conduction tests for carpal tunnel syndrome and tarsal tunnel syndrome protocol were normal, thus excluding large-fiber or compressive neuropathy. Magnetic resonance imaging (MRI) brain and spine screening were normal as well, thus excluding central causes for the symptoms (Figure 2).

An autoimmune neuropathy evaluation panel was performed (Table 3).

**Table 3:** Autoimmune neuropathy panel ELISA: enzyme-linked immunosorbent assay; VGKC: voltage-gated potassium channel; LGI: leucine-rich glioma; CASPR: contactin-associated protein; GAD: glutamic acid decarboxylase.

Serial no	Antibody	Result	Normal reference range
1	Quantitation of neurofascin 140 antibody in the serum by competitive ELISA	63 ng/ml	0-233 ng/ml: negative; ≥234 ng/ml: positive
2	Quantitation of neurofascin 155 antibody in the serum by competitive ELISA	84 ng/ml	0 to 232 ng/ml: negative; ≥233 ng/ml: positive
3	Quantitation of neurofascin 186 antibody in serum by competitive ELISA	623 ng/ml	0 to 366 ng/ml: negative; ≥367 ng/ml: positive
4	Quantitation of myelin-associated glycoprotein antibody in serum by sandwich ELISA	115 ng/ml	0 to 1,230 ng/ml: negative; ≥1,230 ng/ml: positive
5	Neuronal antibody detection in serum by tissue-based indirect immunofluorescence assay	Negative	Negative
6	VGKC-associated LGI-1 and CASPR-2 antibody detection by cell-based indirect immunofluorescence assay	Negative	Negative
7	GAD65 antibody quantitation in serum	<5 IU/ml	0-10 IU/ml: negative

The patient was initially started on 1 g pulse IVIG followed by tapering steroids. The autoimmune neuropathy evaluation panel showed a marked increase in the neurofascin 186 antibody titer (623 ng/ml).

The patient presented on March 15, 2025, with severe, disabling erythromelalgia and erythrodysesthesia. Since she benefited only to a limited extent from corticosteroid therapy, she was counseled about rituximab and was subsequently initiated on 500 mg intravenous rituximab, which she continued to receive until March 19. She was discharged the following day. She returned for her second dose of rituximab in September 2025. Upon presentation, there was marked improvement in her symptoms, including erythrodysesthesia, and on examination, her lower limb power was normal (5/5). 

## Discussion

The report demonstrates a case of neurofascin 186 antibody-positive neuropathy in a 46-year-old female patient. Neuropathies with nodal and paranodal involvement were initially classified as chronic inflammatory demyelinating polyneuropathy according to the Peripheral Nerve Society 2010 criteria. However, later studies revealed that the mechanism and symptoms of these conditions differ, so these neuropathies were described as autoimmune neuropathy in 2021 [1,2].

In both the central and peripheral nervous systems, myelinated nerves have small gaps between sections of myelin called nodes of Ranvier. These nodes, along with nearby areas called paranodes and juxtaparanodes, have structures and proteins that facilitate the rapid and efficient transmission of nerve signals [3]. A node of Ranvier, then, is the part of a nerve without a myelin sheath covering. Such regions contain numerous voltage-gated sodium channels that are essential for the rapid transmission of electrical signals from one node to the next, a process known as saltatory conduction.

Neurofascin-186 is a key protein in nodes of Ranvier. This protein helps gather and anchor these sodium channels in the appropriate place in the nerve cell membrane. In the peripheral nervous system, neurofascin-186 projects from axons and connects with a protein called gliomedin, which is released by Schwann cells. Inside axons, neurofascin-186 also binds to other important proteins, such as ankyrin G and βIV-spectrin, which help keep all of the components stable and in their correct positions [3,4]. Antibodies targeting neurofascin-186 bind to extracellular immunoglobulin domains common to all neurofascins [5].

The onset of autoimmune nodopathy tends to be acute or subacute for patients in middle age [6], though pediatric cases have also been reported [5]. Patients usually present with asymmetric weakness or numbness, with distal weakness or numbness being the core features. Sensory ataxia, tremor, and central nervous system demyelination have been observed in rare cases [1]. The presence of focal segmental glomerulosclerosis has been reported in other rare cases [7]. Central nervous system (CNS) manifestations such as vision impairments, headaches, and dizziness have also been reported in certain instances [8].

Nerve conduction studies have revealed slow conduction velocities, possibly because of demyelinating changes, as well as conduction blocks in certain instances [5,8]. MRI of patients with CNS involvement has shown increased T2-weighted signals in the cerebellum, cerebral white matter, and brain stem as well as in the meninges and spinal cord [8]. Also, brachial plexus MRI abnormalities have been observed in some cases, in which biopsies showed a significant decrease in both large and small myelinated nerve fibers, but there was no evidence of onion bulb formation, nor were there signs of the infiltration of immune cells such as T-cells, B-cells, or macrophages [1].

The treatment of neurofascin 186 autoimmune nodopathy mainly involves a mixed approach including corticosteroids, IVIG, oral steroids, and other immunosuppressors. Differences in response rates to IVIG treatment may be associated with the specific immunoglobulin subclass involved, especially when IVIG is used alongside corticosteroids. Pediatric cases have responded well to IVIG, but some patients relapsed and required subsequent doses [5].

Plasmapheresis and corticosteroids were not effective for patients with antibodies to pan-NF, but anti-CD20 B-cell therapy with rituximab has proved effective in various cases [1]. A meta-review of 23 studies, including two randomized controlled trials, six prospective studies, and 15 retrospective studies, found that rituximab was effective in 63% of patients with chronic inflammatory demyelinating polyneuropathy, 48% of those with anti-myelin-associated glycoprotein neuropathy, and 96% of those with autoimmune nodopathy [9].

Rituximab is administered as an intravenous infusion over 3-6 hours. Subcutaneous formulations are not currently used in neurological practice. There remains no standardized dosing protocol for neuroinflammatory diseases, and the published regimens are heterogeneous in this regard. The two most widely used schedules are either 375 mg/m² weekly for four weeks or two infusions of 500-1,000 mg administered two weeks apart. Before initiation, contraindications such as active infection, severe immunodeficiency, and cardiac disease must be excluded, and baseline investigations, including full blood count, liver function, immunoglobulin levels, and viral serologies (hepatitis B virus, hepatitis C virus, and HIV), are recommended. On infusion days, premedication with intravenous methylprednisolone (100 mg) is typically administered. The retreatment strategies for relapsing disease vary and may be guided by B-cell repopulation (CD19⁺ or CD19⁺/CD27⁺ counts) or performed at fixed intervals, usually every six months [10].

## Conclusions

This case report highlights an atypical and slowly progressing presentation of autoimmune neuropathy with anti-neurofascin 186 antibodies. The patient presented with bilateral palm and sole dysesthesia experienced for two years. The chronic sensory symptoms without significant motor symptoms posed a significant diagnostic hurdle. The detection of neurofascin 186 antibodies was crucial for the diagnosis and selection of appropriate therapeutic immunotherapy since these patients may respond more favorably to agents such as rituximab or corticosteroids than traditional IVIG therapy. Early recognition and targeted therapeutic methods can alter the disease course and provide relief from the symptoms.
